# Outcome of revision of unicompartmental knee replacement: 1,948 cases from the Australian Orthopaedic Association National Joint Replacement Registry, 1999–2008

**DOI:** 10.3109/17453671003628731

**Published:** 2010-03-31

**Authors:** Jacqueline R Hang, Tyman E Stanford, Stephen E Graves, David C Davidson, Richard N de Steiger, Lisa N Miller

**Affiliations:** ^1^AOA National Joint Replacement Registry; ^2^Data Management and Analysis Centre, University of Adelaide, SAAustralia

## Abstract

**Background and purpose:**

Despite concerns regarding a higher risk of revision, unicompartmental knee arthroplasty (UKA) continues to be used as an alternative to total knee arthroplasty (TKA). There are, however, limited data on the subsequent outcome when a UKA is revised. We examined the survivorship for primary UKA procedures that have been revised.

**Methods:**

We used data from the Australian Orthopaedic Association National Joint Replacement Registry (AOANJRR) to analyze the survivorship of 1,948 revisions of primary UKA reported to the Registry between September 1999 and December 2008. This was compared to the results of revisions of primary TKA reported during the same period where both the femoral and tibial components were revised. The Kaplan-Meier method for modeling survivorship was used.

**Results:**

When a primary UKA was revised to another UKA (both major and minor revisions), it had a cumulative per cent revision (CPR) of 28 and 30 at 3 years, respectively. The CPR at 3 years when a UKA was converted to a TKA was 10. This is similar to the 3-year CPR (12) found earlier for primary TKA where both the femoral and tibial components were revised.

**Interpretation:**

When a UKA requires revision, the best outcome is achieved when it is converted to a TKA. This procedure does, however, have a major risk of re-revision, which is similar to the risk of re-revision of a primary TKA that has had both the femoral and tibial components revised.

## Introduction

Unicompartmental knee arthroplasty (UKA) has been performed as an alternative to high tibial osteotomy and total knee arthroplasty (TKA) for unicompartmental arthritis since the 1970s (Marmor 1973). Reported outcomes of UKA vary, with 10-year survivorship ranging from 98% (Berger et al. 1999) to 68% (Rand and Bryan 1988). Currently, the accepted 10-year survivorship is approximately 90% with a revision rate that is twice as high as that of TKA (Knutson et al. 1994, Furnes et al. 2007). In addition, the Australian Orthopaedic Association National Joint Replacement Registry (AOANJRR) has reported for a number of years that the risk of revision is very much dependent on age at the time of the primary UKA, with younger patients having a higher risk of revision (AOANJRR Annual Report 2009).

A major rationale for the use of UKA is its potential as a less invasive temporizing procedure that allows revision to TKA when failure eventually occurs (Marmor 1988, McAuley et al. 2001, Engh 2002, Newman et al. 2009). Advocates of UKA have described its revision as less complicated than revision of TKA (McAuley et al. 2001, Newman et al. 2009) with acceptable survivorship following revision (Lai and Rand 1993, McAuley et al. 2001, Johnson et al. 2007, Dudley et al. 2008).

There are few data on the outcome of revisions of primary UKA. Most studies investigating the results have been small, with less than 80 revisions performed (Barrett and Scott 1987, Padgett et al. 1991, Johnson et al. 2007, Dudley et al. 2008). The only analysis involving a large number of revision procedures (1,135) was published by the Swedish Knee Arthroplasty study (Lewold et al. 1998). It was reported that conversion of a UKA to a TKA had a subsequent re-revision rate of 7% at 5 years. The authors described a comparable 5-year cumulative per cent revision of 4 for primary TKA.

The AOANJRR has been collecting data since 1999, and has recorded almost 2,000 revisions of primary UKA procedures (excluding revision for infection) prior to the December 31, 2008. We used these data to report the early to medium-term outcome of revision of modern UKA prostheses.

## Methods

The AOANJRR commenced data collection on September 1, 1999. This was implemented in a staged manner, becoming fully national in 2002. All hospitals undertaking joint replacement surgery contribute data to the registry. Cross-validation of procedures reported to the registry with government separation data ensures that almost all arthroplasty procedures are recorded by the registry. For this report, the registry analyzed the risk of re-revision of 1,948 first-revision procedures of primary UKA. The data analyzed were recorded by the AOANJRR up to the end of 2008.

The results of this analysis were compared to the outcomes of 896 first revisions of primary TKA. These revisions involved replacement of both the femoral and tibial components. Only first revisions that were undertaken for reasons other than infection were included in this analysis.

The registry classifies revisions as being major or minor. A major revision involves revision of one or more major components. A major component is defined as one that interfaces with bone (with the exception of the patella), either the femoral or the tibial component. Minor revisions are all other revisions. When a UKA is revised, it may involve revision of one or more of the unicompartmental knee components or it may be converted to a TKA.

Patient demographics, reasons for the first revision, and subsequent re-revision rates were determined.

### Statistics

The cumulative per cent revision (CPR) was estimated using the Kaplan-Meier method. Unadjusted CPRs are reported with 95% confidence intervals. Cox proportional hazards models adjusting for age and sex were used to compare revision rates. For each model, the assumption of proportional hazards was checked analytically. If the interaction between the predictor and the log of time was significant in the standard Cox model, then a time varying model was estimated. Time points were selected based on the greatest change in hazard, weighted by a function of events. Time points were iteratively chosen until the assumption of proportionality was met; then the hazard ratios were calculated for each selected time period. In our results, if no time period is specified then the hazard ratio is over the entire follow-up period. Adjustment for bilaterality was not performed, as no bias in including bilateral replacements could be expected (Robertsson and Ranstam 2003). All tests were two-tailed at the 5% level of significance. Analysis was performed using SAS software version 9.2 (SAS Institute Inc., Cary, NC).

## Results

The sex distribution of patients undergoing first revision of primary UKA and TKA reported to the registry was similar. The mean age of patients undergoing revision of primary UKA was 2 years younger than that of patients undergoing revision of primary TKA (Table 1).

**Table 1.** Age and sex distribution of subjects by type of primary knee replacement at the time of revision of the primary

“Revision of primary” by sex		Age (years) ^a^					
	n (%)	Q1	Median	Q3	Mean	SD
UKA	Male	980 (48)	58	64	72	65	9.9
	Female	1,072 (52)	57	65	73	65	10
TKA	Male	2,521 (47)	61	67	74	67	10
	Female	2,885 (53)	61	68	75	68	10
Total		7,458	59	67	74	67	10
^a^ Q1, Q3 are first and third quartiles of the age distribution.						

The indications for revision are listed in Table 2. The most common diagnosis for revision of both primary UKA and TKA was loosening/lysis, representing 50% and 32% of revisions, respectively (Table 2). As expected, revision for progression of disease was more common following primary UKA (17% as compared to 0.6% in the primary TKA group). The proportion of revisions with a diagnosis of infection differed between these groups, with 5.1% of primary UKAs being revised for infection as compared to 23% of primary TKAs. As previously mentioned, however, all patients revised for infection were excluded from further analysis.

**Table 2.** Indications for revision of primary UKA and TKA

	UKA		TKA	
Revision diagnosis	n	%	n	%
Loosening/Lysis	1,035	50	1,707	32
Infection	104	5.1	1,253	23
Pain	254	12	500	9.2
Patello femoral pain	13	0.6	705	13
Progression of disease	354	17	32	0.6
Other	292	14	1,209	22
Total	2,052	100	5,406	100

When a primary UKA was revised to another UKA, the risk of revision was high (Table 3). There was no statistically significant difference if the UKA to UKA revision was minor (usually insert only) or major (UKA to UKA minor vs. UKA to UKA major). For the entire period, Adj HR = 1.3 (95% CI: 0.83–1.9) (p = 0.3).

**Table 3.** Annual cumulative percent revision of “revision of primary” knee replacement (excluding infection) (95% CI)

CPR	1 year	2 years	3 years	4 years	5 years
UKA to UKA minor	16 (11–24)	29 (21–38)	30 (22–40)		
UKA to UKA major	11 (6.8–16)	23 (17–30)	28 (22–36)	31 (24–39)	
UKA to TKA	3.0 (2.2–4.0)	7.1 (5.8–8.7)	10 (8.3–12)	13 (11–15)	15 (12–18)
TKA to TKA	3.6 (2.5–5.2)	8.0 (6.1–10)	12 (9.0–15)	14 (11–18)	18 (14–22)

**Figure 1.**
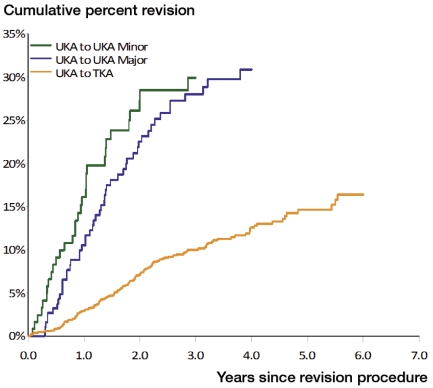
Cumulative percent revision of “revision of primary” UKA (excluding infection).

Conversion of a primary UKA to a TKA had a statistically significantly lower risk of revision compared to both major and minor UKA to UKA revision (Figure 1).

UKA to UKA minor vs. UKA to TKA for 0–1.5 years: HR= 4.8 (3.1–7.5), p < 0.001; for > 1.5 years: HR = 1.8 (0.95–3.6), p = 0.07. UKA to UKA major vs. UKA to TKA for entire period: HR = 2.6 (1.9–3.6), p < 0.001. UKA to UKA minor vs. UKA to UKA major for entire period: HR = 1.3 (0.83–1.9), p = 0.3. Note: adjusted for age and sex.

**Figure 2.**
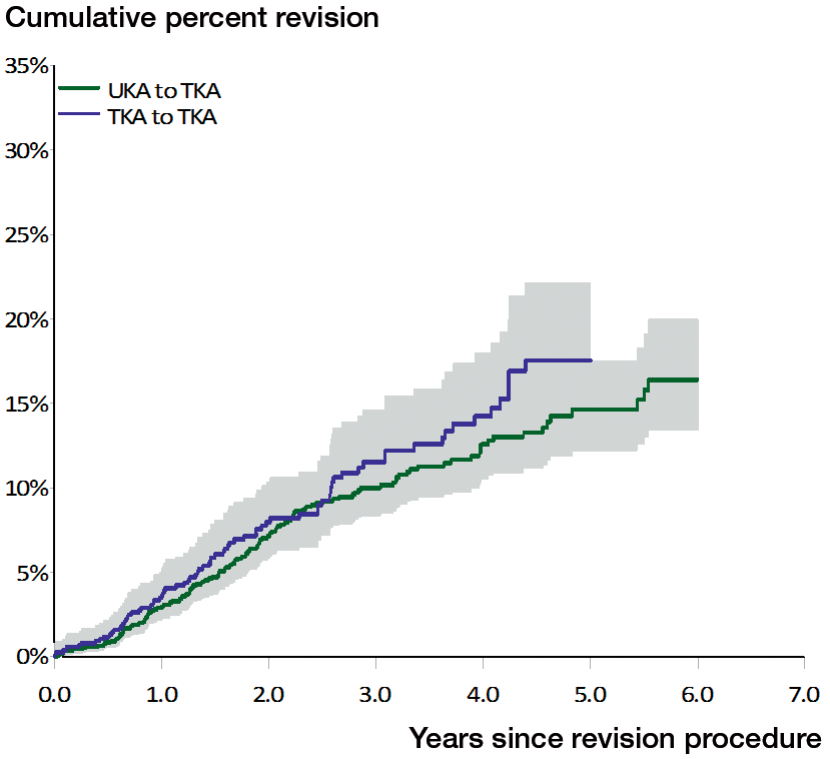
Cumulative percent revision of “revision of primary” knee replacement (excluding infection).

The risk of re-revision when converting a primary UKA to TKA was not statistically significantly different to revision of a primary TKA where both the femoral and tibial components were revised (Figure 2).

TKA to TKA vs. UKA to TKA for entire period: HR = 1.3 (0.95–1.6), p = 0.1. Note: adjusted for age and sex.

The most common indication for re-revision of any type of revision of a primary UKA was loosening/lysis. This was also true for re-revision of revised primary TKA where both the femoral and tibial components were revised (Table 4).

**Table 4.** “Revision of primary” knee replacement: re-revision diagnosis

Re-revision diagnosis	UKA to UKA minor		UKA to UKA major		UKA to TKA		TKA to TKA	
	n	%	n	%	n	%	n	%
Loosening/lysis	17	46	27	52	63	46	34	42
Infection	2	5.4	5	9.6	19	14	25	31
Pain	6	16.2	6	11.5	18	13	8	9.9
Patello femoral pain	1	2.7	1	1.9	9	6.5	3	3.7
Progression of disease	2	5.4	7	14	3	2.2		
Other	9	24.3	6	12	26	19	11	13
Total	37	100	52	100	138	100	81	100

## Discussion

The reported advantages of UKA over TKA include reduced recovery time, greater range of motion, improved gait, increased patient satisfaction, superior preservation of bone stock, and ease of revision (Cameron and Jung 1988, Kozinn and Scott 1989, Lai and Rand 1993, Levine et al. 1996, Chakrabarty et al. 1998, Springer et al. 2006). These factors, as well as good reported survival rates, have led to the belief that should a revision be required, a standard TKA can be performed with high expectations that the outcome will be similar to that of a primary TKA in terms of function and survivorship (Johnson et al. 2007).

Except in a very select number of cases of early revision for failure of fixation where conditions are still optimum for unicompartmental arthroplasty, it is generally accepted practice to revise a failed UKA to a TKA (McAuley et al. 2001). Most published studies on revision of UKA include only revisions to TKA (Barrett and Scott 1987, Padgett et al. 1991, Johnson et al. 2007, Dudley et al. 2008). Apart from the Swedish Knee Arthroplasty study, there are insufficient data in the literature regarding the results of UKA to UKA revision (Lai and Rand 1993, McAuley et al. 2001).

The AOANJRR data indicate that if a primary UKA requires revision for reasons other than infection, then the best outcome is achieved by conversion to a TKA. UKA to UKA revision had a higher risk of re-revision than conversion of a UKA to a TKA. There was a significant difference for both a UKA to UKA minor revision and a UKA to UKA major revision. This difference between UKA to UKA revision and conversion to TKA was also reported by the Swedish Knee Arthroplasty study (Lewold et al. 1998). Unlike that study, however, the AOANJRR data indicate that the risk of re-revision of a UKA converted to a TKA is higher than the outcome of a primary TKA (AOANJRR Annual Report 2009).

The AOANJRR has reported that primary TKA has a CPR of 3.8 at 5 years. Our analysis has shown that the conversion of a UKA to TKA has a re-revision CPR of 15 at 5 years, which is similar to the outcome of a revision of a primary TKA where both the femoral and tibial components have been replaced—rather than the outcome of a primary TKA. It is unclear why this result is different to that reported by the Swedish Knee Arthroplasty study.

It is true that a primary UKA is not as major a procedure as a primary TKA, and that it may provide better clinical outcome and patient satisfaction in the short term. The established higher risk of revision in primary UKA has been accepted largely because it was thought that conversion to a TKA would have a similar outcome to that for a primary TKA. Our large, national analysis indicates that this rationale may have to be reconsidered.
